# Account of the Rise, Progress, and Treatment, of a Fever in the Neighbourhood of Ipswich

**Published:** 1817-06

**Authors:** W. Hamilton


*For the London Medical and Physical Journal.*

### *Account of the Rise, Progress, and Treatment, of a Fever in the Neighbourhood of Ipswich.* By W. Hamilton, M.D.

IN confirmation of the improved practice and excellent rules laid down by Dr. Armstrong, in his late valuable work on Typhus and other Febrile Diseases, I beg leave to state a few cases of that destructive malady, which has frequently made its appearance in this neighbourhood during the last two years.

In the spring of 1816, there were several cases occurred at Whitton, a village about two miles distant from this town, where, in one family, two died.—Mrs. W. the mother of three children, and her husband's brother, who occasionally visited the house while she was confined. These cases had the earliest regular medical attendance. The symptoms were, continued fever, with considerable pain of the head; skin dry, with much thirst; bowels pretty regular, generally too laxative; little sleep and frequent delirium in the night.

 In

In this situation, the unhappy sufferers lingered four or five weeks, becoming weaker every day, and, towards the close of the scene, colliquative diarrhœa and uninterrupted stupor were most conspicuous.

It is almost useless to add, that all the usual remedies were had recourse to, as purging, sudorifics, salines, blisters, and, latterly, cordials and anodynes, without the least beneficial effects.

Three having now died in this situation in the adjoining houses, and the fourth, the father of six children, appearing rapidly to be following the same fatal course, it was determined to change the plan; and, after having bled him to the amount of ten or a dozen ounces, with a view to relieve the head, and finding the bowels generally regular without aperients, the antiphlogistic and sudorific plan was abandoned, and two grains of calomel, with one of opium, were taken three times a day, with two drams of ungt. hydr. rubbed in over the glandular parts night and morning; with an intention to produce ptyalism as soon as possible, as the only means of arresting the progress of the disease. This plan, which was in direct opposition to the advice of some of the medical gentlemen who visited him, succeeded in producing the desired effect on the fourth day. During this interval, I watched the patient with much solicitude, as he seemed every hour to be preponderating between life and death, till I saw with peculiar pleasure the saliva begin to flow, when he was immediately pronounced out of danger. The quantity of calomel was about thirty grains in the four days, with an ounce and a half of blue ointment. The ptyalism, which was rather severe, continued for eight or ten days after; during which time, his only medicine was the acidulated drink, prepared of muriatic acid and sugar, which had the best effect in restoring his strength. This case, being the first recovery from the fever in this village, excited not a little surprise; and soon after another case of an old pensioner occurred, in which, however, from his being already exhausted from the duration of the disease, the mercurial plan was attempted in vain as he died on the second day after, before it could possibly take any effect. This man had lingered for five or six weeks, and had been bled, blistered, and sweated, as well as the bowels kept regular, without the least beneficial result.

The next cases, in March 1817, which came under my notice, were a family of the name of Overton, in which six took the disease, two of whom had it most severely. It was introduced into this family by the eldest daughter, a Mrs. Smith, coming on a visit to her mother, where she was confined fined for ten days or a fortnight with the milder species of this complaint. Her child also had this disease rather severely, but recovered. As soon as she was capable of being removed, she returned to her own home, and scarcely had a day passed before two of her sisters, one about eleven and the other nine years of age, whom she kept, were seized in the night with violent pain in the head and delirium, which was soon succeeded by continued fever and great prostration of strength. This complaint appeared principally seated in the brain, as its chief symptoms were stupor and uneasiness. Their official medical attendant gave them great attention, supplying them well with fever powders, and keeping their bowels laxative with aperients; still, however, they gradually became worse under this treatment, and, about the end of the third week, were so far reduced as to lose their speech, left off eating, and appeared in a constant comatose state, with their eyes scarcely sensible of light, and their frames reduced to mere skeletons. They were now given over, and nothing ordered except wine and water for their drink, as it seemed almost impossible for them to exist much longer. Their poor, but distracted, parents, finding they still survived, on the evening of the subsequent day, applied to me, as they said, merely for satisfaction, as they entertained no hopes of their recovery. The foregoing must suffice for a history of the situation in which I found them, as it would be impossible to paint their melancholy condition in colors sufficiently descriptive of their hapless condition—stretched on the bed of affliction; incapable of either speech or motion; countenance ghastly; eyes insensible; and mouth and tongue dark and parched, with furred lips. In addition to this, the elder was covered with petechiæ, which the next day were succeeded by large vesicles of serum, as if blisters had been applied all over the body. These vesicles subsided in about twenty-four hours, and, when amendment subsequently took place, the cuticle peeled off in large pieces, or, to use the poor woman's phrase, like a snake casting its skin. They were also, at this time, much affected with colliquative diarrhœa, screaming out occasionally from pain in the abdomen, then relapsing into their usual comatose state. It appeared of little use to order any thing internally, as they could scarcely swallow a tea-spoonful of any fluid at once, and, as I expected they would not be alive in the morning, I only blistered the back of their necks, and had their heads repeatedly washed with vinegar and water, as well as gave them a few drops of laudanum on sugar, with a view to still their

2 bowels, bowels, then left them to the protection of Heaven. On visiting them next day, I was not, however, a little surprised to find them not only alive, but considerably revived, the blisters having drawn well, and the bowels being much more still. This favorable aspect encouraged me to farther attempts for their recovery, and I now began the calomel in one-grain powders twice a-day, gave them the acidulated mixture, with just sufficient tinct. opii to prevent its griping or laxative effects; had the rooms well ventilated, and their heads and extremities repeatedly sponged. By a perseverance in this plan for a few days, evident amendment took place, and the submuriate was lessened to one grain every other night, their bowels kept pretty regular with opium, and the mixture continued. From this time, they continued gradually to recover their speech and strength; and, in the course of a few weeks, I had the satisfaction to see them completely restored, by these simple means, to perfect health. In neither of these cases did the effect of the calomel appear on the mouth, owing, no doubt, to their youthful age, although they took more than twenty grains each in less than as many days. The other two brothers, who had the fever at the same time, were affected in a much milder manner, and required only a few doses of submuriate for their recovery. As soon as this family recovered, their next neighbour took the complaint; and here, the father and mother had the milder species of the fever, without any of their children being affected with it. In several other instances, which have lately occurred of this fever in the neighbourhood, this plan of treatment has generally been found sufficient to arrest the progress of the complaint; and I have not known any deaths from it for the last month or two, although it continues occasionally still to make its appearance here; and, from the manner in which it generally runs through the family, there can be but little doubt of its infectious nature.

Since writing the above, which I meant for your last Number, I perceive, both by the public papers, as well as your Journal, that a fever something similar has made its appearance in several other parts of the country: your readers will, no doubt, be much obliged to your numerous correspondents for some further authentic accounts of this complaint.

*Ipswich; May* 2, 1817.

